# Interventions for lifestyle changes to promote weight reduction, a randomized controlled trial in primary health care

**DOI:** 10.1186/1756-0500-6-213

**Published:** 2013-05-27

**Authors:** Stefan PO Jansson, Peter Engfeldt, Anders Magnuson, Georg Lohse PT, Göran Liljegren

**Affiliations:** 1 Family Medicine Research Centre, Örebro County Council, School of Health and Medical Sciences, Örebro University, Box 1613, Örebro, 701 16, Sweden; 2 Centre for Assessment of Medical Technology in Örebro (CAMTÖ), Örebro, Sweden; 3 Department of Surgery, University Hospital, Örebro, Sweden; 4 Clinical Epidemiology and Biostatistics Unit, Örebro University Hospital, Örebro, Sweden

**Keywords:** Lifestyle changes, Obesity, Weight reduction, Family practice

## Abstract

**Background:**

Overweight and obesity are growing public health problems in high income countries and is now growing at a dramatic pace in low and middle income countries, particularly in urban settings. The aim of this trial was to examine the effects of a weight reduction program in adults and to determine whether or not a more extensive intervention was superior to ordinary care.

**Methods:**

Patients seeking advice for overweight/obesity or illness related to overweight/obesity at eight primary health care centers in Sweden were randomized either to intervention or control care groups with both groups given dietary advice and individualized information on increased regular physical activity. In the intervention group advice was more extensive and follow-up more frequent than in the control group during the study period of two years. Main outcome measure was reduction in body weight of five percent or more from study start.

**Results:**

From October 2004 to April 2006, 133 patients, 67 in the intervention group and 66 in the control group, were randomized over a period of 18 months. Target weight was achieved at 12 months by 26.7% of the patients in the intervention group compared with 18.4% in the control group (p = 0.335). There was an average absolute weight loss of 2.5 kg in the intervention group and 0.8 kg in the control group at 12 months as compared with the weight at study entry. There were no significant differences between the groups in quality of life, blood glucose and lipids. At 24 months target weight was achieved in 21.9% versus 15.6%, with an average weight reduction of 1.9 kg and 1.2 kg in the two groups, respectively.

**Conclusions:**

Promotion of a diet with limited energy intake, appropriate composition of food and increased physical activity had limited effects on body weight in a Swedish primary care setting. More extensive advice and more frequent visits made no significant difference to the outcome.

**Trial registration:**

ClinicalTrial.gov: NCT01606917

## Background

Overweight and obesity are growing public health problems in high income countries and is now growing at a dramatic pace in low and middle income countries, particularly in urban settings [1]. Overweight and obesity are major risk factors for a number of chronic diseases, including diabetes, cardiovascular diseases (CVD), musculoskeletal disorders and cancer [2]. These diseases also often have strongly negative effects on quality of life. In Sweden, two million people are overweight and 500,000 are obese (both adults and children) [3,4]. The causes of obesity are only partly known, but genetic factors play a key role [3-5]. Although inheritance plays an important part, obesity is a complex disorder influenced by other factors including diet, exercise, social, behavioral, cultural, and community factors [6,7]. The economic burden of obesity is escalating. For instance, current costs in United States (2010) are estimated to be between $147-$210 billion for obesity-related diseases [8].

Scientific assessments of treatment methods for overweight and obesity have shown modest but not sustained weight reduction over a short period, and the types of weight-loss interventions that contribute most to successful long-term outcomes have not yet been established [9]. Many individuals with overweight /obesity have their health related problems treated by primary care providers. This could also be an opportunity for providers to give patients structured advice about or interventions to combat their overweight/obesity. For severely obese individuals surgical treatment has been the most successful treatment in achieving sustained weight reduction [10]. Regardless of the method used, interventions related to other risk factors can reduce CVD risk, even when weight reduction does not succeed [11]. Recently, a Swedish study in a primary health care setting using lifestyle modifications also showed reduced cardiovascular risk factor levels but not weight loss [12]. Another obesity-related risk factor is type 2 diabetes and for this condition physical activity with even small and moderate weight reductions have been shown to decrease the risk of developing type 2 diabetes [13].

The aim of this two years study was to examine the short and long-term effects of a weight intervention program in adults, seen in a primary care setting. A prerequisite for inclusion was that the patients consulted or were in care for overweight/obesity. The aim was also to compare more extensive advices with appointments and telephone contacts with ordinary care, all done with a pragmatic study design and with limited resources.

## Methods

### Patients

Adult patients between 18 and 70 years of age who consulted or were in care for overweight/obesity with or without type 2 diabetes, hypertension, CVD, coronary heart disease (CHD), dyslipidemia, gallstone, or musculoskeletal disorders at eight Primary Health Care Centers (PHCC) in Örebro County, Sweden were eligible for the trial.

Patients were not eligible if they were already taking part in another weight control program, understood the Swedish language poorly, were mentally ill, or had an alcohol or drug abuse problem. Neither were they eligible if they had a physical disability preventing intensified physical activity or were pregnant at study start.

In the waiting room of the PHCCs, information about the study was posted, inviting patients to take part in the trial. At their appointments with the GPs at the centers, patients were invited to participate in the trial if appropriate, irrespective of whether or not the patients asked about the study. After obtaining informed consent, an individualized target weight was formulated. It was also decided over what time period it should be achieved, the same for both groups. Patients were advised that a realistic goal was a weight reduction of at least 5% by one year after study start.

**Figure 1:**
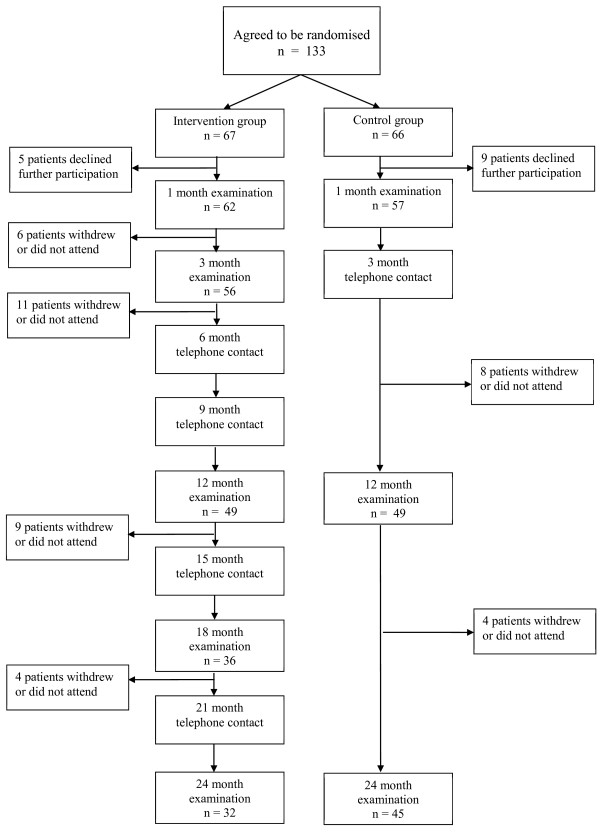
Participants flow diagram.

The patients were then randomized to the intervention or control group. Figure 1 shows the flow diagram for the patients as regards examinations and drop-outs.

### Interventions

Patients in the intervention group had regular appointments five times over the first two years with both a study nurse and a study physiotherapist. In addition, the study nurse and the physiotherapist contacted the patient by telephone four times during study months 6, 9, 15 and 21 (Figure 1). This contact was to encourage patients to comply with the advice given and answer patient questions. At the appointments with the nurse, written and illustrated information of the “plate model” was distributed to the patients and the content described in detail. Moreover, questions were answered and food advice repeated. They were also given a diary in which their physical activity was to be recorded and handed over to the physiotherapist at the check-ups. At the appointments with the physiotherapist a personalized program of regular exercise was designed and continuously adjusted for each participant. At these appointments the study nurse checked blood pressure, height, weight, waist circumference, calculated BMI, and performed blood tests for estimation of glucose and lipid levels. The basis of energy restriction given was the “plate-model”, well known in Sweden, which illustrates the relative proportions of different food groups, in relation to which food of adequate composition and amount was demonstrated to the patient [14]. The model emphasizes that the two main daily meals (lunch and dinner) should contain no more than 25% meat, fish, chicken, eggs, beans, or other vegetarian protein alternatives. The rest of each meal should contain 25% potatoes, pasta or bread and 50% vegetables or fruit. It was recommended that water be the meal time drink. At breakfast, a sandwich and a bowl of yoghurt along with tea or coffee was recommended. Between meals, a fruit or a small sandwich was allowed [15]. This gives an energy intake where 10-15% comes from protein, no more than 30% from fat and the rest from carbohydrates. The advice emphasized an important aspect of the “plate model”, namely to limit the amount of food at each meal. No second helpings or snacks between meals were allowed.

In the control group the ordinary information used at the PHCC by members of the ordinary staff (doctor, nurse and physiotherapist) on the importance of a diet of adequate composition, reducing the total energy intake, and regular physical activity for weight control was given. Food advice was also based on the “plate-model” with the same composition as in the intervention group. The written information on the “plate-model” was given to the patients with no further discussion on the content. Patients in the control group had a check-up with a nurse after one month of compliance with the advice, and a repetition of what had been said at the start. At three months the nurse and the physiotherapist phoned the patients to encourage patients to comply with the advice given. They were also given a diary in which their physical activity was to be recorded and shown to the physiotherapist at the check-ups (Figure 1).

The trial was executed only by staff members at the participating PCHCs who all had a short instruction session by a dietitian before study start to describe the “plate model”, and the effects and importance of physical activity according to the metabolic equivalent (MET) intensities model by a physiotherapist [16]. In both groups, quality of life were estimated at start, after 12 and 24 months using the Short Form 36 items (SF-36) and EuroQol 5-D (EQ-5D) questionnaires. The ordinary equipment such as scales, blood pressure gauge, etc., in place at the different PCHCs were used. No behavioral therapy or motivational interviewing was used in either group.

Biannual meetings, lasting half a day, were organized for the staff members by the steering committee. The purpose of the meetings was to discuss problems that had arisen during the study, but it was also an opportunity for the steering committee to motivate the members to keep up their involvement in the study.

The primary outcome was the proportion of patients who achieved target weight at two years. Secondary outcomes were weight loss, clinical or laboratory manifestations of their baseline illness (type 2 diabetes, hypertension, CHD, dyslipidemia) or death of any cause. Further secondary endpoints were quality of life estimated using the SF-36 and EQ-5D forms. The trial was approved by the regional ethics committee in Uppsala, Sweden (204: M-300) and registered in the Current Controlled Trials, NCT01606917.

### Randomization

After written informed consent, randomization was carried out using numbered sealed opaque envelopes stratified for each PHCC, BMI >30 and type 2 diabetes. The order of the envelopes was predetermined by the study statistician and kept in a box of 20 numbered envelopes of varying bloc sizes at each PHCC by the study coordinator at each centre. At randomization an envelope was drawn in the predetermined numbered order.

### Statistics

SPSS for Windows, version 17.0 (Chicago, IL) was used for all statistical analyses. On the assumption that 50% of the patients in the intervention group and 20% in the control group would achieve their target weight at two years with a power of 90% at a significance level of 0.05, two-sided test, it was determined that 50 patients in each group would have to be included. We estimated that as many as 50% of the patients would have difficulties in fully complying with the study protocol. We therefore aimed to randomize 75 patients to each study group. The analysis was per protocol. Mean with standard deviation (SD), median with quartile range and proportion of individuals was used to summarize different variables. For comparisons between intervention and control groups, chi-square test or Fischer’s exact test when appropriate were assessed for categorical variables, and unpaired and paired t-tests were used for continuous variables with 95% confidence intervals. P-values less than 5% (two-sided) were regarded as statistically significant.

## Results

From October 2004 to April 2006, 133 patients were randomized, 67 to the intervention group and 66 to the control group. The goal of 150 randomized patients could not be reached because of declining interest at the PHCCs in carrying on with inclusion of patients in the trial. Inclusion was therefore stopped on April 30, 2006. Baseline characteristics of the patients are given in Table 1. No apparent differences were observed between the groups, except that there were more men in the intervention group and more patients with CHD in the control group.

**Table 1:** Baseline characteristics among patients

	**Intervention group**	**Control group**
n Age, mean (SD)	67 45 (13)	66 49 (13)
Male/female (% male)	22/45 (32.8)	15/51 (22.7)
Initial weight, kg, mean (SD)	97.7 (13.7)	95.0 (13.4)
BMI, mean	33.8	33.6
Smoker (n)	10	10
Diabetes (n)	8	10
Hypertension (n)	23	26
Dyslipidemia (n)	22	22
Coronary heart disease (n)	1	5
Arthrosis (n)	23	27

There were considerable difficulties for both the primary care organisation and the patients in complying with the study protocol. Therefore only 45 patients in the intervention group and 49 patients in the control group contributed information at 12 months. At that time, 26.7% of the patients in the intervention group had a weight reduction of 5% or more as compared with 18.4% in the control group, Table 2. This difference was not statistically significant (p = 0.335). The corresponding results at 24 months were 21.9% and 15.6%, respectively (p = 0.479). As seen in Table 3, there was an absolute weight loss of 2.5 kg in the intervention group at 12 months as compared with the initial weight at study entry. The corresponding weight loss in the control group was 0.8 kg. The difference between the groups was not statistically significant, -1.7 kg (95% CI -4 to 0.4 p = 0.108). At 24 months the number of patients contributing to the analysis had further declined in the intervention group to 32 patients while still 45 patients were in the control group. At that time the weight loss in each group was 1.9 kg and 1.2 kg, respectively, which was not statistically significant, -0.7 kg (95% CI -3.4 to 1.9, p = 0.572).

**Table 2:** Proportion of patients achieving target weight (minimum 5% weight loss) at 12 and 24 months

	**Intervention group n (%)**	**Control group n (%)**	**p**
Achieved target weight at 12 months	12 (26.6)	9 (18.3)	0.335
Achieved target weight at 24 months	7 (21.9)	7 (15.6)	0.479

**Table 3:** Weight loss at 12 and 24 months, comparisons between and within groups

	**Intervention group mean (95% CI) n = 45 at 12 months n = 32 at 24 months**	**Control group mean (95% CI) n = 49 at 12 months n = 45 at 24 months**	**Mean weight difference between groups at 12 and 24 months (95% CI)**	**p**
Weight change at 12 months, kg	-2.5 (-4.0 to -1.0)	-0.8 (-2.3 to 0.8)	-1.7 (-3.8 to 0.4)	0.108
Weight change at 24 months, kg	-1.9 (-4.0 to 0.2)	-1.2 (-2.9 to 0.6)	-0.7 (-3.4 to 1.9)	0.572

There was significant weight loss in the intervention group at 12 months as compared within the group (95% CI -4.0 to -1.0, p = 0.001). At 24 months this difference was no longer significant (95% CI -4.0 to 0.2, p = 0.07), Table 3.

There were no effects on plasma glucose, blood lipids or blood pressure between the study start and values at 12 and 24 months (data not shown). Quality of life was assessed using both the SF-36 (data not shown) and the EQ-5D form, Table 4. No statistically significant difference on these items was noticed over the first 12 months of the trial. Too few measurements of quality of life were made at 24 months to make any comparison meaningful. Low compliance with the activity diary in both groups made it impossible to evaluate the intensity of physical activity.

**Table 4:** EQ5-D. Proportion of patients with fewer symptoms and better performance at 12 months than at baseline

**Question**	**Improved, intervention group, n**	**Improved, control group, n**	**p**
Motility (%)	42 (7)	35 (9)	0.571
Hygiene (%)	41 (2)	36 (3)	>0.999
Main activities (%)	43 (14)	35 (9)	0.504
Pain/inconvenience (%)	39 (13)	34 (24)	0.358
Anxiety/depression (%)	41 (15)	36 (17)	0.999
Health barometer (%)	42 (48)	36 (61)	0.262

## Discussion

The main finding of this trial showed that intervention was not superior to ordinary advice to promote weight reduction. Although the difference between the groups was not statistically significant in terms of the primary outcome, patients in both groups had weight loss of between 1.9 and 1.2 kg even after two years of follow-up.

The magnitude of weight loss of 2.5 kg at 12 months in the intervention group seems mostly to have been maintained over the next 12 months. The narrowly missing significance (p = 0.07) may be attributed to the smaller than planned sample size owing to high drop-out rates. The weight loss of 3% is comparable to what can be achieved using drugs such as orlistat and sibutramin [3], the latter withdrawn from the market in 2010. The same weight loss was also attained for 29,560 adults referred to Weight Watchers by the National Health Service (NHS) [17]. The maintenance of the initial weight reduction at two years is encouraging, especially because even small and moderate weight reductions have been shown to decrease the risk of developing type 2 diabetes, one of the obesity-related risk factors [18]. The decrease in weight loss in the intervention group between 12 and 24 months was probably an effect of the well-known difficulties in maintaining a lifestyle change over time [19] as well as that attendance at face-to-face counseling sessions decreases substantially over time [20]. One interesting observation was that the control group had a trend of catching up with the intervention group and seemed to find it easier to comply with the treatment regime, since they had a higher participation rate. It might be more feasible to have fewer appointments at PHCCs and supportive telephone contact between appointments for participants, as in the control group, than to follow the regimen used in the intervention group. Furthermore, many individuals todays find an intervention such as this study difficult to fit in with all their other activities, despite the awareness that their health would benefit, which limits their attendance rate. Other unknown factors may also have contributed to the small differences between study groups seen at the study end. Further key factors that have an influence on how successful individuals are in weight loss and maintenance of new weight, include psychosocial variables concerning exercise and eating behaviors. This was tested in a study of overweight and obese women who underwent a behavioral obesity treatment program of 12 months, with a further 12 months (non-intervention) of follow-up. The results showed that lowering emotional eating and adopting a flexible dietary restraint pattern were critical for sustained weight loss [21]. Our study did not comprise such factors.

Scientific assessments of treatment methods for overweight and obesity have only shown modest but not sustained weight reduction over a short period [9], although they are highly cost-effective in primary care models [22].

A short study from eight primary care practices in the UK reported a weight loss of 4.0 kg in the intervention group as compared with 1.2 kg in the ordinary care group over 12 weeks in a weight management programme. The conclusions drawn from that study were that it was feasible to follow the NICE guidelines [23]. Overall, very little research has been conducted on the management of obesity in primary care practice, as was recently concluded in a systematic review [24], in which the best results of weight loss intervention were achieved with primary care providers plus pharmacotherapy or intensive counseling plus meal replacements. More recently, another study concluded that it is not known how effective weight loss interventions may be in real world situations such as clinical or community practice settings [25].

Physicians’ recommendations have a strong impact on individual health behaviors [26] and physicians have good opportunities to help their patients further when the patients present for overweight/obesity related problems. However, a recent survey among primary care physicians concluded that less than 10% reported always referring their patients with weight related diseases for further management [27]. Further research is needed to identify barriers to providing care and to improving physicians’ engagement in managing healthy lifestyles in adults. It might be better to involve other providers than primary care based services to overcome these barriers. For instance in one study, comparing eight different weight loss programmes of 12 weeks duration, it was concluded that commercially provided weight management services were more effective and cheaper than primary care based services [28].

The Swedish Björknäs Study [12] performed at one primary health care centre managed to keep their patients in the trial over a three-year period, with a withdrawal rate of approximately 10-15%. The aim of the lifestyle intervention was to lower cardiovascular risk factor levels. They found statistically significant improvements in most of their estimated parameters except for blood lipids and plasma glucose. Our aim was a weight loss of at least 5% compared with the weight at study start. While a weight reduction was achieved in our trial this was not the case in the Björknäs study. However, they found a reduction in waist circumference and waist-hip ratio.

From the perspective of most patients with overweight or obesity, a weight reduction of 2.5 kg is disappointing. Bearing in mind that it takes time to become overweight or obese it is not surprising that weight reduction also takes time. This is a considerable challenge to any clinician who sees such patients, especially when bariatric surgery is so successful in reducing weight, both in a short and a long-term perspective [10]. Access to bariatric surgery is limited and most obese patients have to wait to have the operation performed. During that period, lifestyle changes like those in this trial have no obvious drawbacks, but do have the potential to make obese patients more fit for the surgery. In this study, just under one fifth of the patients achieved weight loss of at least 5% of their baseline weight. Two other studies recently reported that more than one third of the patients responded to lifestyle counseling with weight loss of 5% [29,30]. Since many obese patients do not achieve sufficient weight loss to have a meaningful impact on their health with lifestyle changes alone, other options must be offered, for instance, bariatric surgery.

The small effects on weight did not impact on any aspects of quality of life as estimated using SF-36 and EQ-5D. This is not surprising, bearing in mind the size of the effect, the difficulties in executing the protocol over time, and that only measurement at 12 months could be analyzed.

### Strengths and limitations

The strength of our study was that it was an attempt to perform a study in a routine clinical setting with no extra resources. The limitations include the difficulty of carrying out a trial in such a setting in an organization with a staff with high turnover and with shortages of GPs and other professionals. This influences the continuity and the involvement of the staff members. The regular meetings for the staff members held twice a year could not counteract this negative progress. The importance of this is illustrated in the Björknäs study, in which one committed person managed to keep almost all the patients in the study throughout the whole study period [12]. Furthermore, the staff members and participants were not blinded to the intervention. Perhaps, if the nurses had been blinded to the outcome (weight), their motivation might have been better to encourage the study participants to make the necessary lifestyle changes. This could have led to bias.

The high drop-out rate in our trial is a major drawback to the internal validity of the trial results at two years. However, the results of this trial probably reflect what is achievable in a Swedish primary care setting without extra added resources. The drop-out rate in this study, 56 participants (42%), is higher than in two other two-year studies, 5%, and 14%, respectively [29,30]. On the other hand, follow-up data at one year from another study showed a 30% drop-out rate [28]. The relatively high drop-out rate in this study could introduce a selective bias, i.e. patients in the intervention group who failed to lose weight, could have had less motivation to carry on in the study. Moreover, reduced sample sizes at study end result in a loss of power.

## Conclusions

A multimodal intervention to promote a diet with limited energy intake and increased physical activity had limited effects on body weight among patients in a Swedish primary care setting. More extensive advice and more frequent visits made no significant difference to the outcome.

## Competing interests

None of the authors has any conflicts of interest concerning the interventions and performance of this trial. The authors alone are responsible for the content and writing of the paper.

## Authors’ contributions

The study was conceived and designed by SJ, PE and GL who also wrote the manuscript. SJ, PE, AM and GL analysed and interpreted data. SJ, PE, AM, GeL and GL reviewed and edited the manuscript. All authors read and approved the final manuscript.
